# Protocol for reference-free single-cell-resolution deconvolution of spot-based spatial transcriptomics with CoexpressDeconvolve

**DOI:** 10.1016/j.xpro.2026.104947

**Published:** 2026-11-05

**Authors:** Olga Perik-Zavodskaia, Roman Perik-Zavodskii, Saleh Alrhmoun, Sergey Sennikov

**Affiliations:** 1 Laboratory of Molecular Immunology, Research Institute of Fundamental and Clinical Immunology, 630099 Novosibirsk, Novosibirsk Region, Russia

**Keywords:** Bioinformatics, Single Cell, Cancer, Genomics, RNAseq

## Abstract

Spot-based spatial transcriptomics captures whole-transcriptome signal per spot but mixes multiple cells. Here, we present a protocol for using CoexpressDeconvolve, a reference-free approach that reconstructs single-cell-resolution, whole-transcriptome profiles directly from spot-based spatial transcriptomics data. We describe steps for housekeeping-gene cell-density calibration, joint overdispersed-gene selection, a gene co-expression manifold, and automated topic-number selection. We then detail procedures for latent Dirichlet allocation, hierarchical Bayesian sampling, and exporting to a 10×-compatible object for standard single-cell and spatial cell-cell communication analysis.

For complete details on the use and execution of this protocol, please refer to Perik-Zavodskaia et al.^1^

## Highlights

• Steps for housekeeping-gene calibration of the number of cells per spot
• Steps for automated topic-number selection and consensus topic-model reconstruction
• Steps for reference-free deconvolution of spot transcriptomes into single cells
• Steps for export to a 10× object and single-cell spatial analysis

## Abstract (editor highlights)

Publisher’s note: Undertaking any experimental protocol requires adherence to local institutional guidelines for laboratory safety and ethics.

## Graphical abstract

**Figure:**
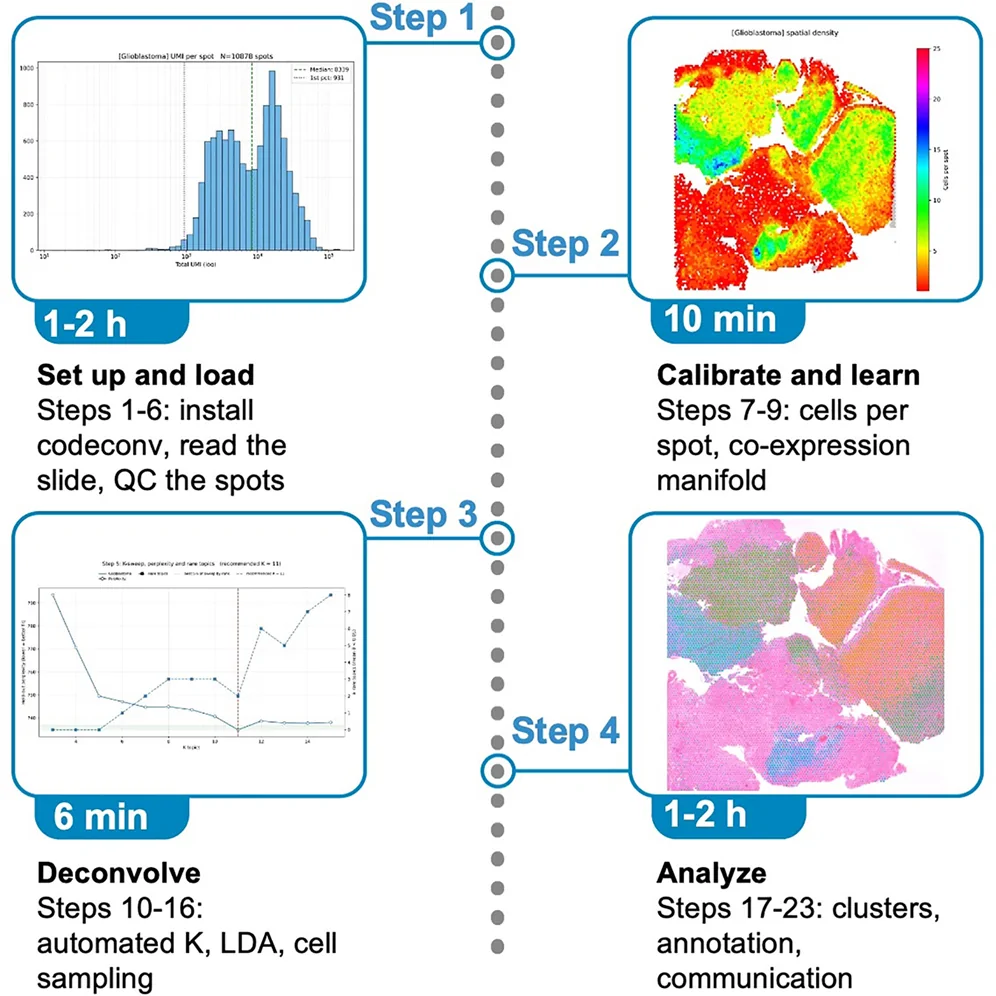

## Before you begin

Spot-based spatial transcriptomics, such as 10× Visium, preserves tissue architecture while providing whole-transcriptome measurements, and it has become central to dissecting tissue ecosystems in health and disease.^2^ However, each Visium spot aggregates transcripts from several neighboring cells, so spot-level profiles are cellular mixtures rather than single cells.

Two families of methods address this. Reference-based tools map an external single-cell atlas onto the spots and return per-spot cell-type proportions; they are accurate when a matched atlas exists, but they cannot recover cell types that are absent from the reference because they were lost during dissociation. Reference-free tools remove that dependency. STdeconvolve^3^ established this direction by fitting latent Dirichlet allocation directly to the spot corpus, and the overdispersed-gene selection used in this protocol follows the same mean-variance criterion. What STdeconvolve returns, however, is a per-spot distribution over topics: proportions over a selected gene set.

CoexpressDeconvolve differs in what it returns rather than in whether a reference is required. It reconstructs discrete, individually placed cells carrying absolute whole-transcriptome counts, written in the 10x SpaceRanger layout, so the output is a single-cell object rather than a proportion table. The two approaches are therefore complementary, and the choice between them follows from the downstream question: proportions per spot, or cells to cluster, annotate and pass to single-cell-resolution tools. A quantitative head-to-head against STdeconvolve, together with the reference-based methods Tangram and cell2location, is reported in the main research article on synthetic Visium data with known ground truth.^1^

The protocol below describes the specific steps used for the large and resource-heavy 11 mm human glioblastoma Visium dataset analyzed here. This dataset is a real-world example rather than a validation experiment: it is a public tumor section with no per-spot cell-type ground truth, chosen because it is realistic, large enough to exercise the memory-limiting steps, and freely available. Quantitative validation of the method, covering both recovery of known cell-type composition and expression fidelity, is reported in the main research article on synthetic Visium data with known composition and on a breast cancer section with a paired single-cell atlas.^1^

### Applicability to other spot-based platforms

The protocol applies unchanged to any 10x Visium sample (6.5 mm or 11 mm, standard or Visium CytAssist) of any organism, provided a species profile is available (see “Select or configure the species profile”). It is not restricted to Visium (Table 1). Everything after data loading needs only four quantities per sample: a gene-by-barcode count matrix, per-barcode coordinates, a capture-unit footprint, and a center-to-center pitch. Step 5 detects the platform from the input layout, normalizes these four quantities, and reports what it found, so no manual conversion is required.

**Table 1:** Spot-based platforms read natively by the protocol

Platform	Capture unit	Pitch	Input layout	Histology
10x Visium (6.5 or 11 mm, CytAssist v2)	55 μm	100 μm	SpaceRanger output: filtered_feature_bc_matrix.h5 (or the matrix folder) and spatial/	Yes
DBiT-seq^4^	10, 25 or 50 μm	2 × the channel width	Single count table on the 50 × 50 microfluidic grid, coordinates in the row labels	No; placeholder generated

Capture units at or below the size of a single cell do not require deconvolution, and the protocol is deliberately not extended to them. Other technologies, such as Slide-seq, Visium HD, Stereo-seq, Xenium, Merfish, and CosMx are subcellular by construction. For such data the appropriate step is segmentation and assignment, not deconvolution, and applying a mixture model to a capture unit that holds at most one cell would impose structure that is not there.

***Note:*** Platforms that ship no histology image receive a black placeholder canvas sized to the capture grid, together with synthesized scale factors, so that Seurat::Load10X_Spatial() and the spatial plotting functions still work. A warning is printed when this happens.

### Innovation

CoexpressDeconvolve reconstructs absolute, whole-transcriptome, single-cell-resolution expression from spot-based spatial transcriptomics without requiring an external single-cell reference atlas. Whereas proportion-based deconvolution methods return per-spot cell-type fractions, CoexpressDeconvolve instead (i) calibrates the number of cells per spot from housekeeping-gene signal blended with UMI depth, (ii) learns a whole-transcriptome co-expression structure de novo via a gene manifold and consensus latent Dirichlet allocation,^5^ and (iii) samples discrete, individually placed in-silico cells through a hierarchical Bayesian engine that conserves per-spot counts. Because no reference is used, the method can recover fragile cell types that are systematically lost during dissociation and are therefore missing from single-cell atlases. The output is written in the standard 10x SpaceRanger HDF5 layout, so the reconstructed cells drop directly into mature single-cell ecosystems (Seurat,^6^ scanpy) and single-cell-resolution spatial tools (CellChat^7^^,^^8^) with no bespoke adapters. This protocol also adds an on-disk (BPCells) execution path so that the tens of thousands of reconstructed cells can be analyzed on a standard workstation.

### Institutional permissions

This protocol uses a publicly available 10x Genomics demonstration dataset (Human Brain Cancer, 11 mm Capture Area, FFPE; CC BY 4.0)^9^ and requires no institutional permissions. Investigators applying the protocol to newly generated patient or animal tissue must obtain the relevant institutional and national approvals before sample collection.

## Key resources table

**Table:** 

REAGENT or RESOURCE	SOURCE	IDENTIFIER
**Deposited data**		

Human Brain Cancer, 11 mm Capture Area (FFPE); Visium CytAssist v2	10x Genomics	https://www.10xgenomics.com/datasets/human-brain-cancer-11-mm-capture-area-ffpe-2-standard (CC BY 4.0)
Worked-example inputs and reconstructed output (this protocol)	This protocol	https://github.com/Perik-Zavodskii/CoexpressDeconvolve/

**Software and algorithms**		

CoexpressDeconvolve (codeconv) v1.0.0	Perik-Zavodskaia et al.^3^	https://pypi.org/project/codeconv/; https://github.com/Perik-Zavodskii/CoexpressDeconvolve; https://doi.org/10.5281/zenodo.21860849
CoexpressDeconvolve Config Maker (species-profile generator)	Perik-Zavodskaia et al.^3^	https://github.com/Perik-Zavodskii/CoexpressDeconvolve
Python ≥3.9 with numpy, pandas, scipy, scikit-learn, umap-learn, h5py, matplotlib, seaborn and tqdm; versions pinned by the codeconv package	Python Software Foundation	RRID:SCR_008394
R 4.5.2	R Core Team	RRID:SCR_001905
Seurat v5.4.0	Hao et al.^6^	RRID:SCR_007322
BPCells 0.3.1	Parks and Greenleaf^10^	https://github.com/bnprks/BPCells
CellChat 2.2.0	Jin et al.^7^^,^^8^	RRID:SCR_021946
ComplexHeatmap 2.26.0	Gu et al.	RRID:SCR_017270
Space Ranger 2.0.1	10× Genomics	RRID:SCR_017344

**Other**		

Workstation with 16 GB RAM and a multi-core CPU (example run: Apple M1 Pro, 16 GB, macOS 26.5 Tahoe)	N/A	N/A

## Materials and equipment

### Hardware and operating system

This protocol targets a 16 GB workstation throughout; the worked example was run on an Apple M1 Pro with 16 GB of unified memory under macOS 26.5 Tahoe, with R 4.5.2. On this dataset (a single 11 mm slice of approximately 10,878 spots and approximately 45,530 reconstructed cells), the deconvolution (Steps 5–16) completes in approximately 15 min end-to-end with a peak resident memory of approximately 3.8 GB. Wall-clock time is dominated by Step 9, the gene co-expression manifold, at approximately 9 min; all other steps take seconds to a few minutes. The downstream Seurat analysis on the reconstructed cells is the memory-limiting stage and relies on the BPCells on-disk path to stay within 16 GB. No GPU is required. Timings and peak RAM are reported per step below and are printed automatically by each step.

The pipeline is pure Python and runs natively on macOS, Linux and Windows; no component is platform-specific. The only package needing attention on Windows is BPCells, which compiles C++ and therefore requires Rtools. Timings on Intel or AMD hardware of comparable core count are similar, because the bottleneck in Step 9 is single-threaded and CPU-bound.

### Key run parameters (this dataset)

The values below reproduce the worked example. Each entry states what the parameter controls, a sensible range, and how to recognize that it is set wrongly (Table 2).

**Table 2:** Key run parameters

Parameter	Value	What it controls, sensible range, and how to tell it is wrong
seed	42	Global random-number seed (codeconv.set_seed). Fixing it makes the topic fit, the sampler draws and the cell placement reproducible. Any integer; change it only to test stability.
species	hs	Which housekeeping genes, noise pattern, marker set and sampler parameters are used. Profiles for human (“hs”) and mouse (“mm”) are built into the module; other organisms need a profile from the Config Maker. A wrong species fails visibly at Step 7, because the housekeeping gene names will not match the matrix.
min_umi	900	UMI floor below which a spot is treated as background and assigned zero cells. Set it near the first percentile of the per-spot UMI histogram from Step 6, which is 931 here. Too high and genuine sparse tissue is discarded; too low and empty resin is populated with cells. Diagnosed on Figure 1A and from the background count printed by Step 7.
anchor_mean_factor	0.7	Scales the housekeeping standard against which per-spot cell number is calibrated. It is an empirical scaling rather than a biological constant: tune it so that the standard line falls on the first non-zero peak of the housekeeping signal (Figure 1B). Typical range 0.5–1.0; raise it for densely cellular tissue and lower it for sparse or degraded FFPE. If most spots collapse to zero or one cell, this is the first parameter to revisit (Troubleshooting problem 2).
low_slice_quality	FALSE	Enables a per-spot floor of one cell and topic rescue for weak sections. Leave it FALSE for good-quality material; set it TRUE when a section is degraded and Step 7 assigns zero cells to spots that clearly contain tissue in the image.
n_odg	3000	How many overdispersed genes enter the topic model. The usual range is 2,000–5,000; the value matters much less than whether the selected genes sit above the mean-variance trend, which Figure 3 shows directly. Too few and rare programs are missed; too many and the corpus fills with noise, which flattens the perplexity curve.
n_components	30	Independent components computed before the UMAP projection that forms the gene manifold. 20–40 works for most tissues. Too few and unrelated genes collapse together; too many and the manifold fragments. Check on Figure 4 that the configured marker genes sit in coherent, separated neighborhoods.
min_k, max_k	3, 15	Range of topic numbers evaluated in the K-sweep. Start max_k at roughly 1.5 times the number of cell populations expected: 15 suits a single tumor section, 20–30 a whole organ or a multi-region sample. Sweep cost grows roughly linearly with the number of K values. A recommendation landing on max_k means perplexity had not started to rise again and the sweep is too narrow (Troubleshooting problem 5).
perc_rare_thresh	0.05	Mean per-spot theta below which a topic is counted as rare. This is a diagnostic only; rare topics are not penalized by the selection rule, because in a cell-generating setting they correspond to minor populations that are worth recovering.
k_band	0.05	Width of the low-perplexity band used by the automated topic-number rule: keep the K values whose held-out perplexity falls in the best 5% of the sweep by rank, and take the largest of them. Lower it for a stricter, smaller K. Above roughly 0.10 the rule begins to admit K values that are visibly worse.
n_topics	11	Final number of topics, taken from the K-sweep recommendation in Step 11 and confirmed against the per-topic markers in Step 13. Too low and distinct lineages merge into a single topic; too high and one lineage splits into near-duplicate topics, which appears as redundant marker lists in model.qc_df (Troubleshooting problem 3).
k_neighbors	3	Number of manifold neighbors used to project the consensus topic-gene matrix onto the full cleaned gene list. This projection is also what allows sparsely detected genes to borrow topic weight from their co-expression neighbors. 3–5 is usual; larger values smooth the projection and blur topic-specific genes.
min_topic_percentage	0.00	Per-spot theta below which a topic is zeroed before sampling. Leave it at 0 to retain rare programs. Raise it only when the sampler produces cells from topics that are not credibly present in a spot.
interaction_range_um	50	Interaction radius in microns, recorded at export for the downstream CellChat analysis. On Visium the 50 μm default is the shortest radius that still spans the whole capture-spot footprint, which is what keeps the communication inference independent of the sub-spot cell placement (see Step 21 and limitations).

## Step-by-step method details

***Note:*** The CoexpressDeconvolve API function names encode pipeline stages (codeconv.step1_acquisition_and_anchoring through codeconv.step9_export_results) and are unrelated to the protocol step numbers used below. Function names are given in full wherever a call is made, so the two numbering schemes cannot be confused.

### Install the software environment

**Timing: 30 min to 1 h (excluding download times)**

1. Install a Python ≥3.9 environment and CoexpressDeconvolve.

pip install codeconv

***Note:*** This installs the codeconv module together with its dependencies (numpy, pandas, scipy, scikit-learn, umap-learn, h5py, matplotlib, seaborn, tqdm). Human and mouse species profiles are built into the module, so nothing else has to be downloaded before the first run. To work from a source checkout instead, clone https://github.com/Perik-Zavodskii/CoexpressDeconvolve (archived at https://doi.org/10.5281/zenodo.21860849), place codeconv.py in the working directory or on the Python path, and install the dependencies with pip.

2. Install R ≥4.3 and the downstream-analysis packages.

install.packages(c("Seurat", "ggplot2", "dplyr", "patchwork", "viridis",

"reshape2", "future", "jsonlite"))

remotes::install_github("bnprks/BPCells/r")

remotes::install_github("jinworks/CellChat")

install.packages("ComplexHeatmap") # or BiocManager::install("ComplexHeatmap")

**CRITICAL:** BPCells is required for the downstream analysis at this cell count; installing Seurat alone will exhaust memory during scaling and PCA on a 16 GB machine. On Windows, BPCells compiles C++ and therefore needs Rtools installed first.

### Obtain the example dataset

**Timing: 10–30 min (network dependent)**

3. Download the Space Ranger outputs for “Human Brain Cancer, 11 mm Capture Area (FFPE)” from 10x Genomics (https://www.10xgenomics.com/datasets/human-brain-cancer-11-mm-capture-area-ffpe-2-standard) and unpack them so that a folder named “Glioblastoma” contains a “filtered_feature_bc_matrix” folder (“filtered_feature_bc_matrix.h5” is also accepted) and a “spatial” folder.

***Note:*** This is a Visium CytAssist v2 (11 mm) FFPE sample processed with Space Ranger 2.0.1; 10,878 spots are detected under tissue, with a median of approximately 8,339 UMIs and approximately 4,600 genes per spot.

### Select or configure the species profile

**Timing: 5 min**

4. Confirm the species profile used for the run. For this dataset set species = “hs” (human); a mouse profile (“mm”) is also built in. Both are used automatically, with no configuration file present. The profile supplies four things:
  a. hk_profiles: housekeeping genes and their expected relative levels, used for cell-density calibration in Step 7.
  b. noise_regex: gene-name pattern removed before feature selection (mitochondrial, ribosomal, lncRNA and similar) in Step 8.
  c. qc_markers: marker genes highlighted on the gene manifold in Step 9.
  d. engine_parameters: the sampling engine’s mu and phi (mean and dispersion of the per-cell library-size distribution) used in Step 14.

***Optional:*** To inspect or hand-tune a profile, write the built-in configuration to a file, edit it, and pass its path as config_path to any step that takes one. An external file always takes precedence over the built-in profiles.

import codeconv

codeconv.write_default_config("codeconv_config.json")

***Optional:*** For organisms other than human or mouse, generate the species block with the CoexpressDeconvolve Config Maker before running (see Troubleshooting problem 1).

jupyter notebook "CoDeconv Config Maker/Estimate single cell parameters.ipynb"

# set species = 'other' and

# datasets_root = <folder of 10x scRNA-seq datasets for the organism>

***Note:*** The Config Maker walks a folder of 10x scRNA-seq datasets for the target organism and writes the species block into codeconv_config.json: hk_profiles are the median per-gene log1p(CP10K) of the housekeeping genes across datasets, and engine_parameters (mu, phi) are estimated from the library-size distribution. An existing species block's gene lists and noise_regex are preserved; only the numerical values are recomputed.

### Load the data and inspect quality

**Timing: <1 min (3.8 s on the example dataset; peak RAM 1.6 GB)**

This step loads the count matrix and coordinates for each slice, detects and reports the source platform, records per-spot UMI totals, and produces a per-slice UMI histogram for quality inspection (Figure 1A).

5. Import the CoexpressDeconvolve module, set the random seed, and define the paths and species.

import codeconv

codeconv.set_seed(42)

spatial_path = "./Glioblastoma"

output_folder = "."

species = "hs"

6. Load the data. Confirm the platform reported on the first line of the output, then inspect the printed median, mean and first-percentile UMI and the per-slice histogram.

**Figure 1:**
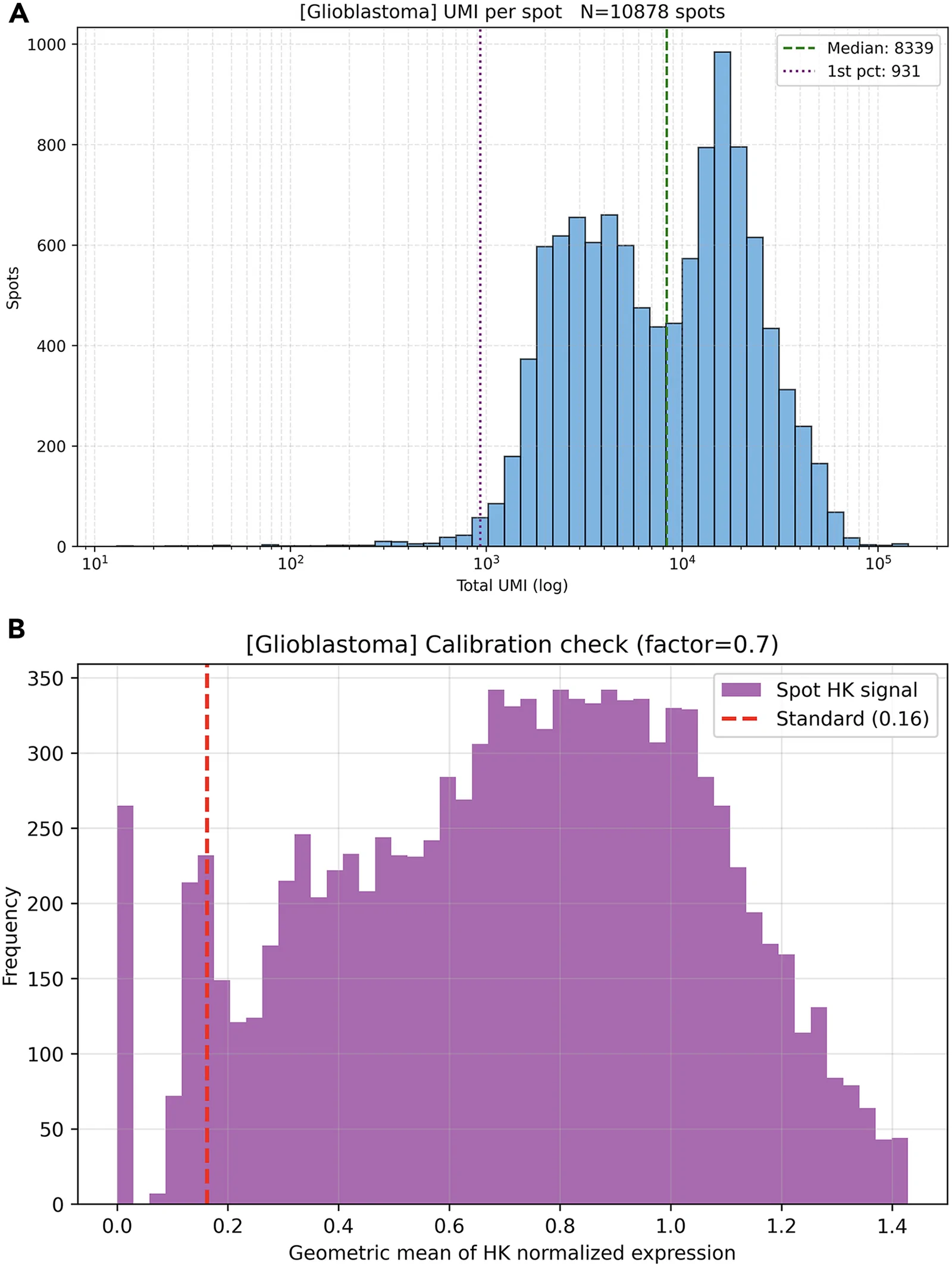
Input quality control and housekeeping calibration (Steps 5–7) (A) Per-spot UMI histogram for the glioblastoma slide (N = 10,878 spots), with the median (8,339) and first-percentile (931) UMI marks. (B) Housekeeping-signal calibration check: the geometric mean of housekeeping-normalized expression per spot against the tissue standard (dashed line) at anchor_mean_factor = 0.7.

slices = codeconv.step1_acquisition_and_anchoring(spatial_path)

***Note:*** spatial_path accepts a string (single slice), a list, or a {name: path} dict for multi-slice runs; per-slice parameters may then be passed as dicts keyed by slice name. The platform is detected from the input layout and printed as, for example, “Detected platform: 10x Visium”. Detection is a heuristic, so pass platform=“visium” or platform=“dbit_seq” to override it.

### Estimate per-spot cell density

**Timing: <1 min (2.0 s on the example dataset; peak RAM 1.3 GB)**

This step calibrates the number of cells per spot by blending housekeeping-gene signal with UMI depth against a tissue standard, and outputs a calibration check (Figure 1B) together with the per-spot cell-count distribution and spatial-density map (Figure 2).

7. Estimate density, adjusting min_umi and anchor_mean_factor to the sample.

slices = codeconv.step2_estimate_cell_density(

slices, species=species,

min_umi=900,

anchor_mean_factor=0.7,

low_slice_quality=False,

colormap='hsv',

)

**CRITICAL:** Every later step inherits this calibration: the number of cells sampled per spot, and therefore the total cell count, follow directly from it. Inspect the calibration histogram before continuing. The red standard line should align with the first non-zero peak of the housekeeping-gene signal (Figure 1B).

***Note:*** anchor_mean_factor is an empirical scaling of the housekeeping standard, not a biological constant. The value of 0.7 used here places the standard on the first non-zero peak for this FFPE section; the usual working range is 0.5–1.0, higher for densely cellular tissue and lower for sparse or degraded material. If most spots collapse to zero or one cell, or too many spots are dropped as background, revisit min_umi and anchor_mean_factor (Troubleshooting problem 2).

**Figure 2:**
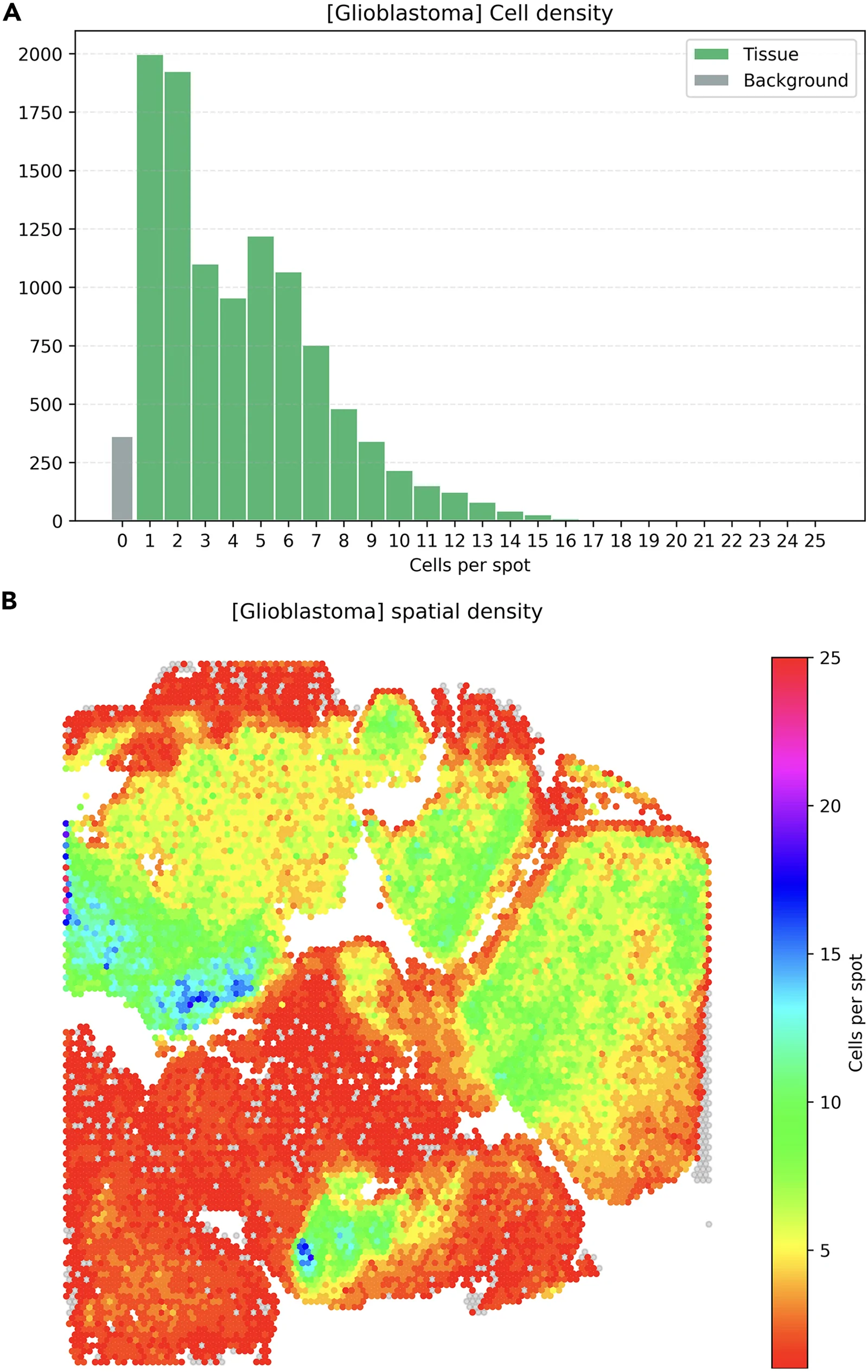
Per-spot cell-density estimation (Step 7) (A) Distribution of the estimated number of cells per spot (tissue spots in green, background or zero-cell spots in gray). (B) Spatial map of estimated cells per spot across the slide.

### Filter noise and select overdispersed genes

**Timing: <1 min (4.5 s on the example dataset; peak RAM 3.5 GB)**

This step removes noise genes by the species regex, intersects genes across slices, restricts candidates by spot-presence fraction, and selects the most overdispersed genes from the mean-variance trend (Figure 3).

8. Run feature selection. The diagnostic plot shows filtered, candidate and selected genes on the overdispersion axis.

odg_pack = codeconv.step3_feature_selection(

slices, species=species,

n_odg=3000,

)

***Note:*** Genes detected in fewer than 5% or more than 95% of spots are excluded before selection. Removing the sparse tail matters for what follows: genes too rarely detected to carry usable co-expression signal are kept out of the manifold rather than contributing noise to it. On this dataset 54 noise genes are removed, leaving 18,031, of which 16,611 pass the presence filter and 3,000 are selected as overdispersed.

**Figure 3:**
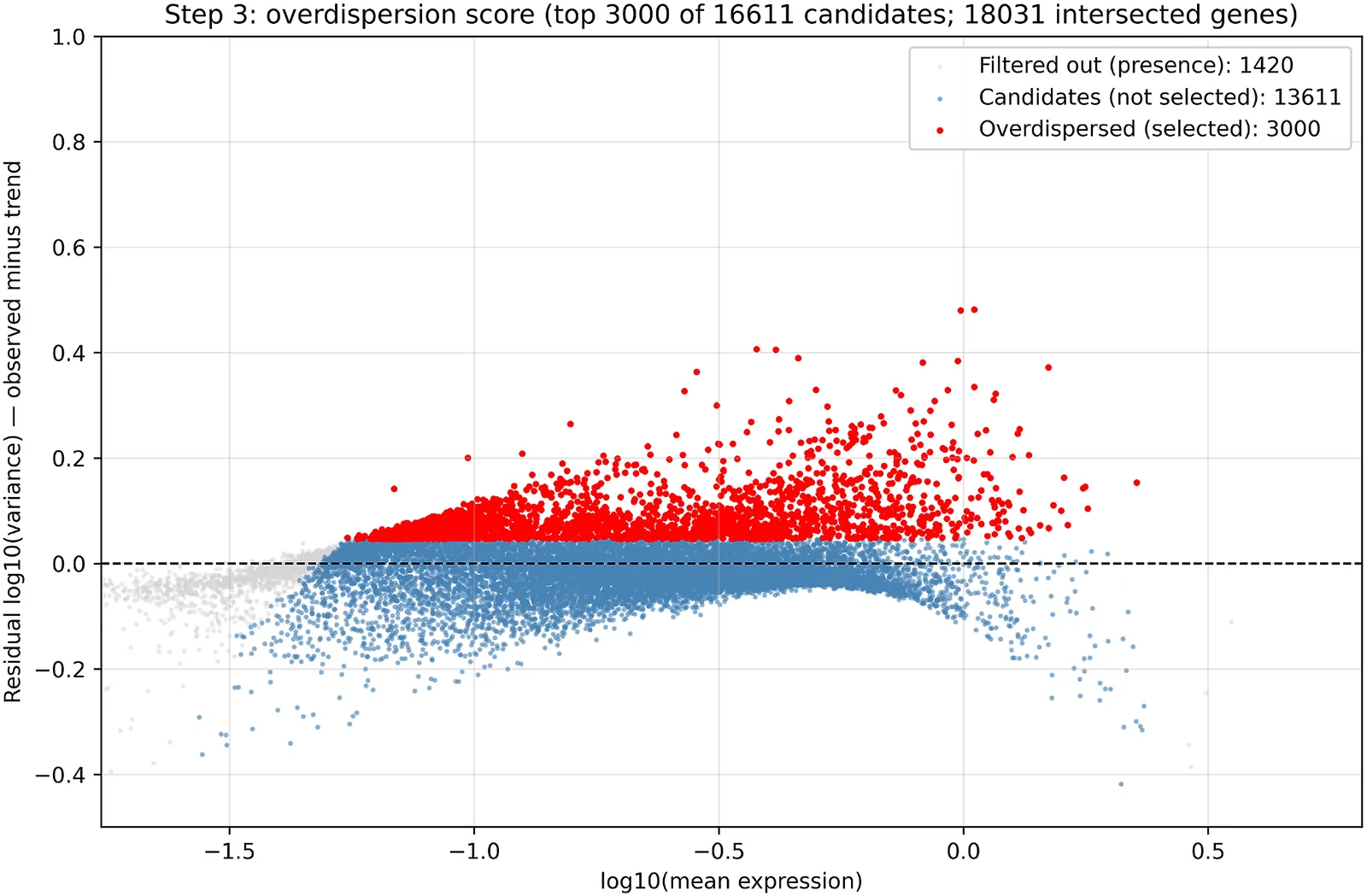
Overdispersed-gene selection (Step 8) Mean-variance overdispersion score for the 18,031 intersected genes; the 3,000 selected overdispersed genes (red) are shown among the 16,611 presence-filtered candidates (blue) and the filtered-out genes (gray).

### Build the gene co-expression manifold

**Timing: Approximately 9 min (533 s on the example dataset; peak RAM 3.6 GB)**

This step embeds genes into a co-expression manifold (independent component analysis followed by UMAP); QC markers from the species profile are highlighted to sanity-check the structure (Figure 4). This is the most computationally intensive step of the pipeline.

9. Build the manifold.

manifold = codeconv.step4_gene_manifold(

slices, odg_pack,

n_components=30,

)

***Note:*** This step dominates run time, at approximately 9 min here against seconds to a few minutes for every other step, because the UMAP embedding^11^ is built over the full cleaned gene set of approximately 18,000 genes. It is CPU-bound and single-threaded; plan the wall-clock budget around it. Each gene is positioned by its profile across all 10,878 spots, so a gene's coordinate is determined by the whole section rather than by any individual spot.

**Pause point:** The manifold and the overdispersed-gene pack are the inputs to everything that follows and can be reused across topic-number choices. Save them if the session is to be interrupted, so that Steps 8 and 9 need not be repeated.

**Figure 4:**
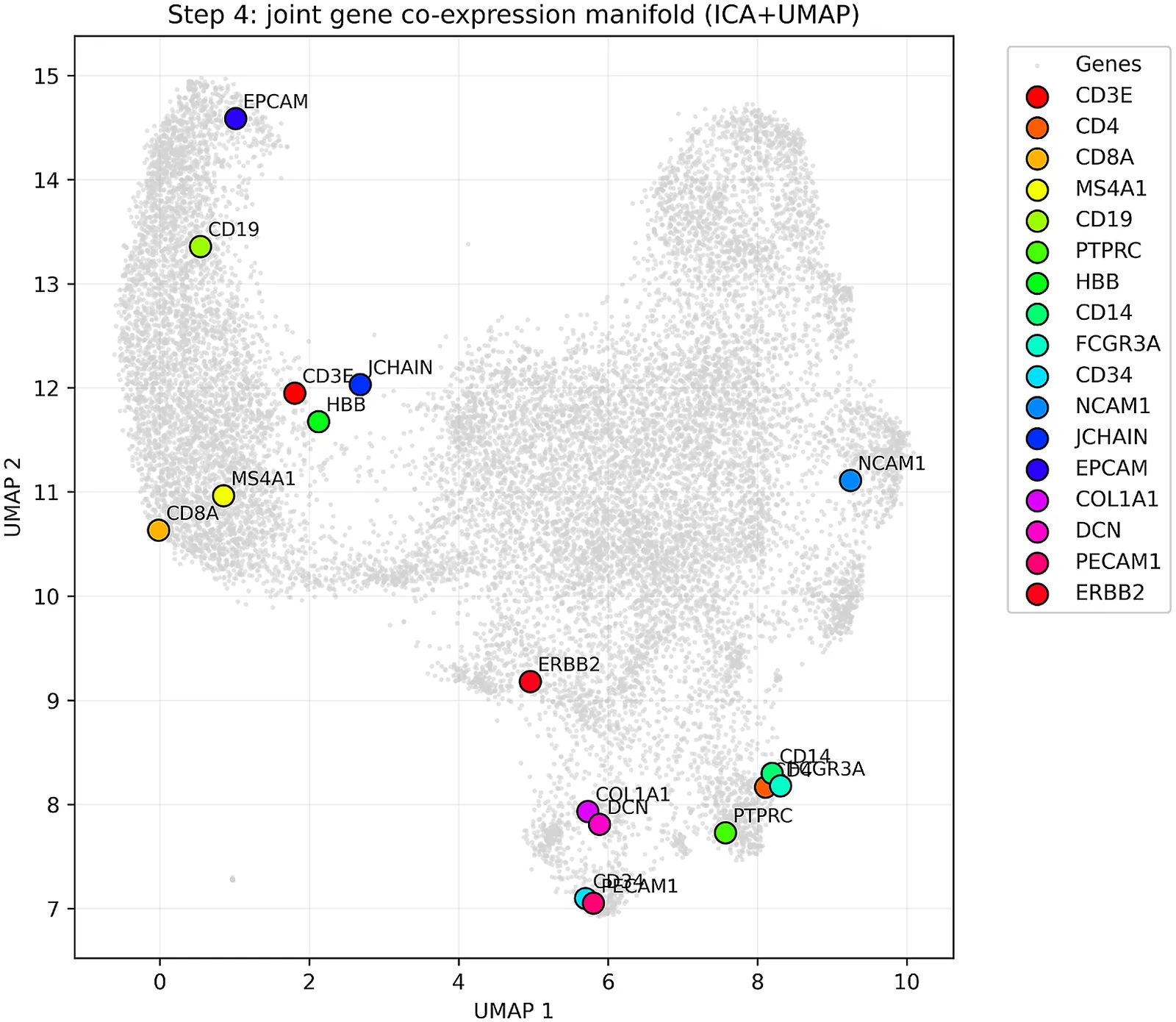
Gene co-expression manifold (Step 9) ICA and UMAP embedding of genes, with the configured QC marker genes highlighted and labeled to sanity-check the manifold structure.

### Select the number of topics by K-sweep

**Timing: Approximately 3–4 min (205 s on the example dataset; peak RAM 2.1 GB; scales with the K range)**

This step fits latent Dirichlet allocation across a range of topic numbers on held-out spots, plots held-out perplexity together with the count of rare topics, and recommends K automatically (Figure 5).

10. Run the sweep.

ksweep = codeconv.step5_ksweep(

odg_pack,

min_k=3, max_k=15,

perc_rare_thresh=0.05,

)

11. Read the recommended K, which is printed by Step 10 and marked on Figure 5, and inspect the per-K metrics behind it.

**Figure 5:**
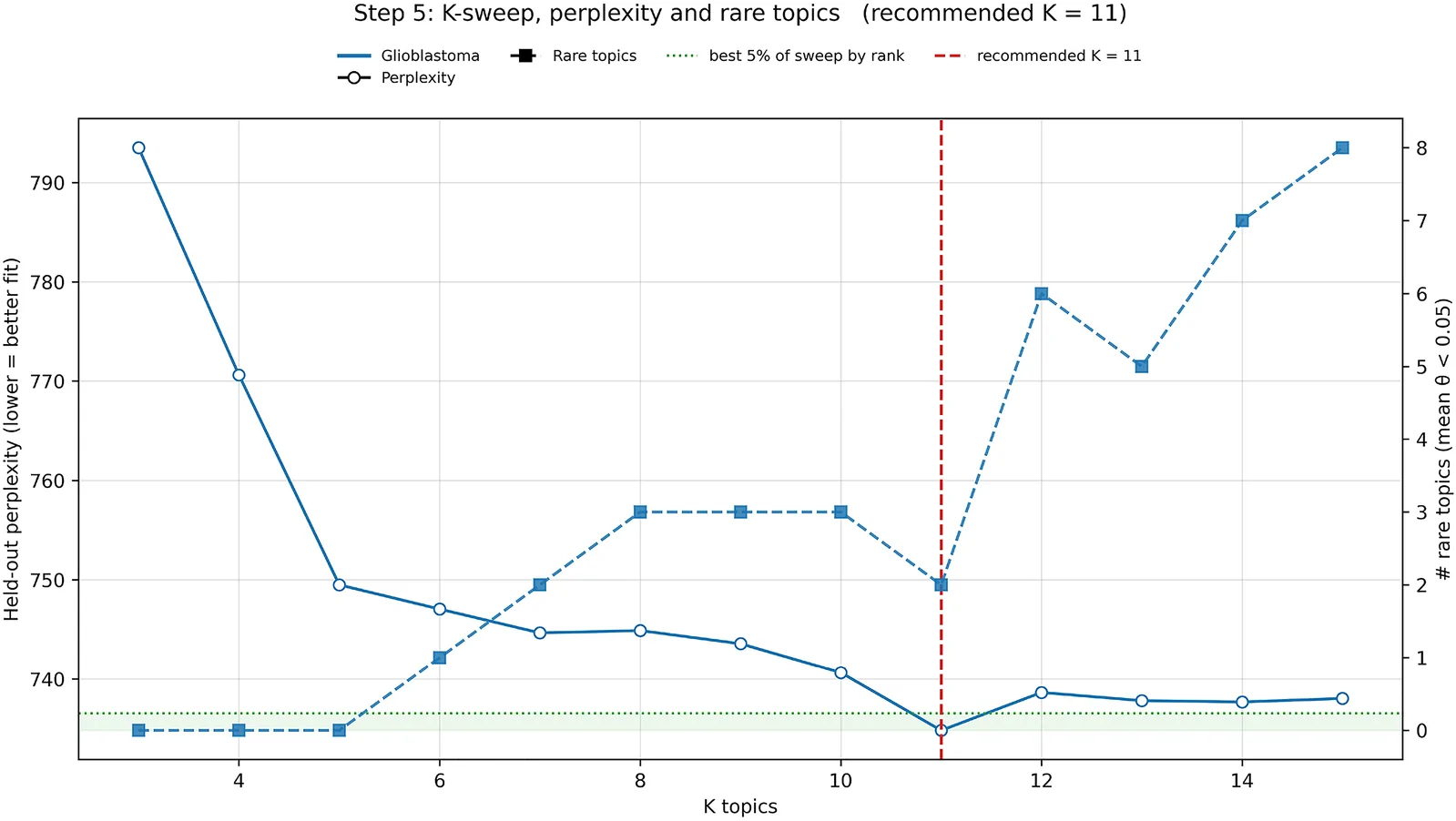
Topic-number selection by K-sweep (Steps 10 and 11) Held-out perplexity (solid, left axis) and the number of rare topics (dashed, right axis) across K = 3–15. The shaded band marks the low-perplexity region used by the selection rule (the best 5% of the sweep by rank) and the vertical line marks the recommended K = 11, the largest K within that band, which is used for the final deconvolution in Step 12.

print(ksweep.k) # recommended number of topics

print(ksweep.summary()) # per-K perplexity, rare topics, band membership

***Note:*** The rule is deliberately not “the K with the lowest perplexity”. Among the K values whose held-out perplexity falls in the best 5–10% of the sweep by rank, the largest is chosen. Unlike proportion-based deconvolution, small topics are desirable here: because CoexpressDeconvolve returns individual single-cell-like profiles rather than fractions, a rare topic corresponds to a genuine minor cell population that should be recovered, so the rare-topic count is diagnostic rather than a penalty. Widen or tighten the band with k_band; k_band = 0.05 is the default. Two alternative rules are available for comparison, k_rule=“relative_tolerance” (every K within 1% of the best perplexity) and k_rule=“lowest_perplexity” (plain minimum). For this dataset the recommendation is K = 11, used in Step 12.

***Note:*** A recommendation equal to max_k triggers a warning: it means perplexity had not started to rise again and the sweep is too narrow to bound K from above. Widen the sweep before accepting the value (Troubleshooting problem 5). To record a manual choice instead, pass n_topics= to codeconv.step5_ksweep; it is drawn on Figure 5 alongside the automatic recommendation, and ksweep.k then returns the manual value.

### Run consensus LDA deconvolution

**Timing: Approximately 2 min (123 s on the example dataset; peak RAM 1.9 GB)**

This step fits latent Dirichlet allocation per slice, aligns topics across slices by Hungarian matching (if multiple slices are being analyzed), forms a consensus topic-gene matrix for multiple similar sections, and projects it onto each slice’s full cleaned gene list. It also reports the top marker genes per topic.

12. Fit the consensus model.

model = codeconv.step6_final_deconvolution(

slices, odg_pack, manifold,

n_topics=ksweep.k,

k_neighbors=3,

)

13. Inspect the top markers per consensus topic.
  display(model.qc_df)

***Note:*** Topics are de novo gene co-expression programs, not annotated cell types. A topic often corresponds one-to-one with a lineage, but it may also split one lineage into states or merge two rare ones, so the downstream inspection in Seurat is essential, where cell-type identities are assigned (Step 19).

***Note:*** The projection onto the full gene list uses the k_neighbors nearest genes on the manifold. This is what carries sparsely detected genes: a gene with few counts still receives topic weight from its co-expression neighbors, so topic profiles are not restricted to well-detected genes.

### Sample *in silico* single cells

**Timing: Approximately 30 s (33 s on the example dataset; peak RAM 3.8 GB)**

This step draws discrete cells per spot through a hierarchical Bayesian engine; topics below min_topic_percentage are zeroed and theta renormalized before sampling.

14. Sample the cells.

cells = codeconv.step7_sampling_engine(

slices, model, species=species,

low_slice_quality=False,

min_topic_percentage=0.00,

)

***Note:*** The sampler works in three stages. Each spot has a topic mixture (theta from Step 12) and a cell count (from Step 7); every cell in the spot is first assigned a topic of origin by drawing from that mixture. Each cell is then given a library size drawn from the species negative-binomial distribution (the mu and phi of the species profile), and these per-cell library sizes are rescaled so that they sum exactly to the UMIs observed in that spot. Finally, the counts of each cell are distributed over genes by a multinomial draw from its topic's projected gene profile. The consequence worth stating plainly is that no UMI is created or destroyed: the reconstructed cells of a spot add back up to the transcriptome that was measured there. The mathematical formulation is given in the main research article.^1^

### Place cells in space

**Timing: <1 min (0.1 s on the example dataset; peak RAM 1.6 GB)**

This step assigns each reconstructed cell a spatial coordinate within the footprint of its parent spot, using an area-uniform Vogel packing.

15. Place the cells.

codeconv.step8_geometry_and_placement(cells, slices)

***Note:*** The packing fills the spot disk uniformly by area and is bounded by the spot radius, so cells never spill into a neighboring spot. The arrangement is geometric rather than measured; the consequences for downstream neighborhood analysis are set out in Step 21 and in the Limitations section.

### Export to 10× format

**Timing: <1 min (0.8 s on the example dataset; peak RAM 1.9 GB)**

This step writes a per-slice 10x Space Ranger HDF5 object plus spatial files, ready for Seurat::Load10X_Spatial(), and records the geometry the downstream CellChat analysis needs.

16. Export the results.

codeconv.step9_export_results(

cells, slices, output_folder,

spot_pitch_um=100.0,

interaction_range_um=50.0,

)

***Note:*** Output is written to slice_<name>/deconvolved/. For this dataset, 45,530 in-silico cells are generated from 10,878 spots across 11 whole-transcriptome projected topics. The per-slice run_summary.json additionally carries a “cellchat” block with the pixel-to-micron ratio, the tolerance, and the interaction and contact ranges, so that the R session in Steps 20–23 reads these values instead of re-deriving them. The default spot_pitch_um of 100 is correct for every Visium capture area; pass the array pitch for other platforms.

**Pause point:** The exported object is self-contained. The downstream analysis can be resumed at any later time, on a different machine, from slice_<name>/deconvolved/ alone.

### Analyze reconstructed cells in Seurat with BPCells

**Timing: 20–60 min (dataset and machine dependent; the memory-limiting stage)**

This step loads the deconvolved object, stores UMI counts on disk with BPCells, and runs a standard single-cell workflow of normalization, PCA, clustering, marker detection, annotation and spatial mapping (Figure 6).

17. Load the deconvolved slice and move counts on-disk with BPCells.

library(Seurat); library(BPCells)

seurat_obj <- Load10X_Spatial("./slice_Glioblastoma/deconvolved")

write_matrix_dir(seurat_obj[["Spatial"]]$counts, dir = "codeconv_counts_bpcells")

seurat_obj[["Spatial"]]$counts <- open_matrix_dir("codeconv_counts_bpcells")

18. Normalize, select variable features, scale, and run PCA, UMAP and clustering.

seurat_obj <- NormalizeData(seurat_obj)

seurat_obj <- FindVariableFeatures(seurat_obj, nfeatures = 3000)

seurat_obj <- ScaleData(seurat_obj) # variable features only

seurat_obj <- RunPCA(seurat_obj, npcs = 30)

seurat_obj <- RunUMAP(seurat_obj, dims = 1:23)

seurat_obj <- FindNeighbors(seurat_obj, dims = 1:23)

seurat_obj <- FindClusters(seurat_obj, resolution = 0.5)

**CRITICAL:** Scale variable features only, which is the Seurat default. Scaling all genes on approximately 45,000 cells builds a dense matrix that exhausts memory even on the BPCells path.

19. Detect markers, assign cell-type identities, and visualize the result.

all_markers <- FindAllMarkers(

seurat_obj,

only.pos = TRUE,

min.pct = 0.25,

logfc.threshold = 0.5

)

library(dplyr)

all_markers_sorted <- all_markers %>%

group_by(cluster) %>%

arrange(cluster, desc(avg_log2FC))

write.csv(all_markers_sorted, file = "all_cluster_markers.csv", row.names = FALSE)

new_cluster_ids <- c(

"0" = "Excitatory cortical neurons",

"3" = "GABAergic interneurons",

"8" = "Neurons",

"10" = "Interneurons",

"4" = "Mature myelinating oligodendrocytes",

"5" = "OPALIN+ mature oligodendrocytes",

"9" = "Reactive oligodendrocytes",

"2" = "NPC-like",

"13" = "OPC-like",

"6" = "MES-like",

"7" = "Tumor-associated microglia",

"11" = "Perivascular stromal cells",

"12" = "Endothelial cells",

"14" = "Dying cells",

"1" = "Dying cells"

)

# Apply to Seurat object

seurat_obj <- RenameIdents(seurat_obj, new_cluster_ids)

seurat_obj$CellType <- Idents(seurat_obj)

DimPlot(seurat_obj, reduction = "umap", repel = TRUE, label = TRUE, pt.size = 0.5)

***Note:*** Annotation must be done on the reconstructed cells, not on the topics.

**Figure 6:**
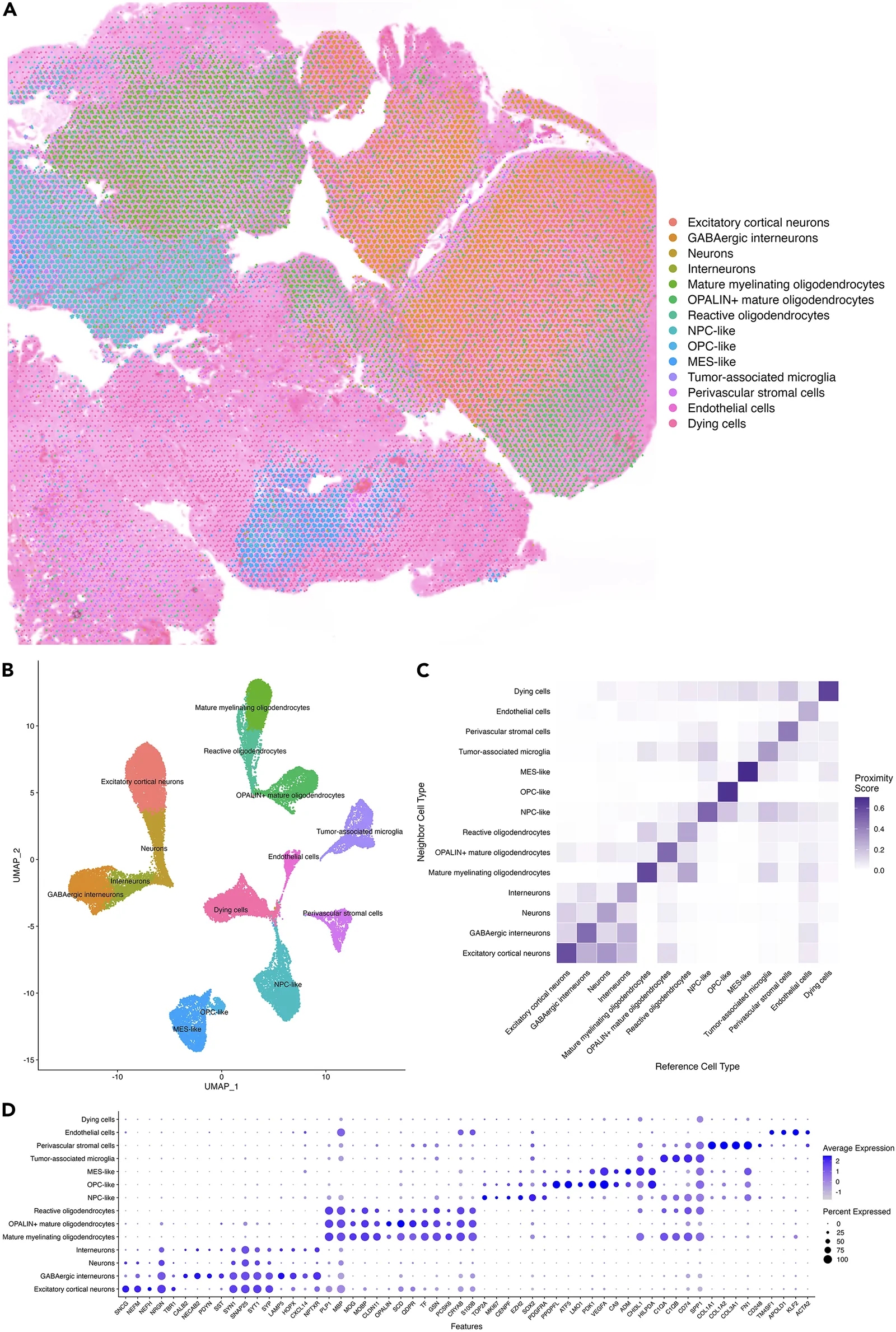
Single-cell-resolution downstream analysis of the reconstructed cells (Steps 17–19) (A) Annotated cell types displayed as a SpatialDimPlot over the tissue image. (B) UMAP of the reconstructed cells colored by annotated cell type. (C) Neighborhood co-localization (proximity) heatmap between reference and neighbor cell types. (D) Marker DotPlot of the genes defining each annotated cell type (average expression and percent expressed).

### Map spatial cell-cell communication with CellChat

**Timing: 15–60 min (distance-constrained inference is compute and memory intensive)**

This step infers ligand-receptor communication on the reconstructed single cells using their spatial coordinates, restricting signaling to a defined interaction distance, and summarizes incoming and outgoing signaling roles as heatmaps (Figure 7).

20. Build the CellChat inputs: normalized expression, cell labels, and pixel coordinates.

library(CellChat); library(ComplexHeatmap); library(jsonlite)

data.input <- as(GetAssayData(seurat_obj, assay = "Spatial",

layer = "data"), "dgCMatrix")

meta <- data.frame(labels = Idents(seurat_obj),

row.names = colnames(seurat_obj))

meta$labels <- droplevels(meta$labels)

spatial.locs <- as.matrix(GetTissueCoordinates(seurat_obj)[, 1:2])

rownames(spatial.locs) <- colnames(seurat_obj)

21. Read the spatial factors recorded at export and derive scale.distance.

cc <- fromJSON("./slice_Glioblastoma/run_summary.json")$cellchat

spatial.factors <- data.frame(ratio = cc$ratio, tol = cc$tol)

cellchat <- createCellChat(object = data.input, meta = meta,

group.by = "labels", datatype = "spatial",

coordinates = spatial.locs,

spatial.factors = spatial.factors)

cellchat@DB <- CellChatDB.human # CellChatDB.mouse for mouse samples

meta.t <- data.frame(group = cellchat@idents,

samples = cellchat@meta$samples,

row.names = rownames(cellchat@meta))

res <- computeRegionDistance(coordinates = spatial.locs, meta = meta.t,

interaction.range = cc$interaction_range,

ratio = cc$ratio, tol = cc$tol,

contact.dependent = TRUE,

contact.range = cc$contact_range)

d <- res$d.spatial

diag(d) <- NA # excluded, as computeCommunProb does

d <- d[is.finite(d) & d > 0] # drop non-adjacent group pairs

scale.distance <- 1.5 / min(d)

***Note:*** CellChat requires the minimum scaled distance to fall in [1, 2] and stops with an error if it does not.

22. Run communication inference under the recorded interaction distance.

cellchat <- subsetData(cellchat)

cellchat <- identifyOverExpressedGenes(cellchat)

cellchat <- identifyOverExpressedInteractions(cellchat)

cellchat <- computeCommunProb(cellchat, type = "truncatedMean", trim = 0.1,

distance.use = TRUE,

interaction.range = cc$interaction_range,

scale.distance = scale.distance,

contact.dependent = TRUE,

contact.range = cc$contact_range)

cellchat <- filterCommunication(cellchat, min.cells = 10)

cellchat <- computeCommunProbPathway(cellchat)

cellchat <- aggregateNet(cellchat)

**CRITICAL:** The interaction range determines whether the result reflects tissue architecture or the sub-spot packing of Step 15. It must be large enough to span the whole capture-spot footprint; the 50 μm default satisfies this on Visium, where the footprint is approximately 60 μm. Step 16 reports the fraction of within-spot cell pairs captured at the chosen range and warns below 95%. See Limitations.

23. Compute network centrality and draw the incoming and outgoing signaling-role heatmaps.

cellchat <- netAnalysis_computeCentrality(cellchat, slot.name = "netP")

ht_out <- netAnalysis_signalingRole_heatmap(cellchat, pattern = "outgoing")

ht_in <- netAnalysis_signalingRole_heatmap(cellchat, pattern = "incoming")

# Both heatmaps are named "Relative strength" by default, which collides

# on draw(); rename them before combining.

ht_out@name <- "Outgoing"; ht_out@matrix_color_mapping@name <- "Outgoing"

ht_in@name <- "Incoming"; ht_in@matrix_color_mapping@name <- "Incoming"

draw(ht_out + ht_in, ht_gap = unit(1, "cm"))

***Note:*** CellChatDB.human is used for this dataset; select CellChatDB.human or CellChatDB.mouse to match the sample's species.

**Figure 7:**
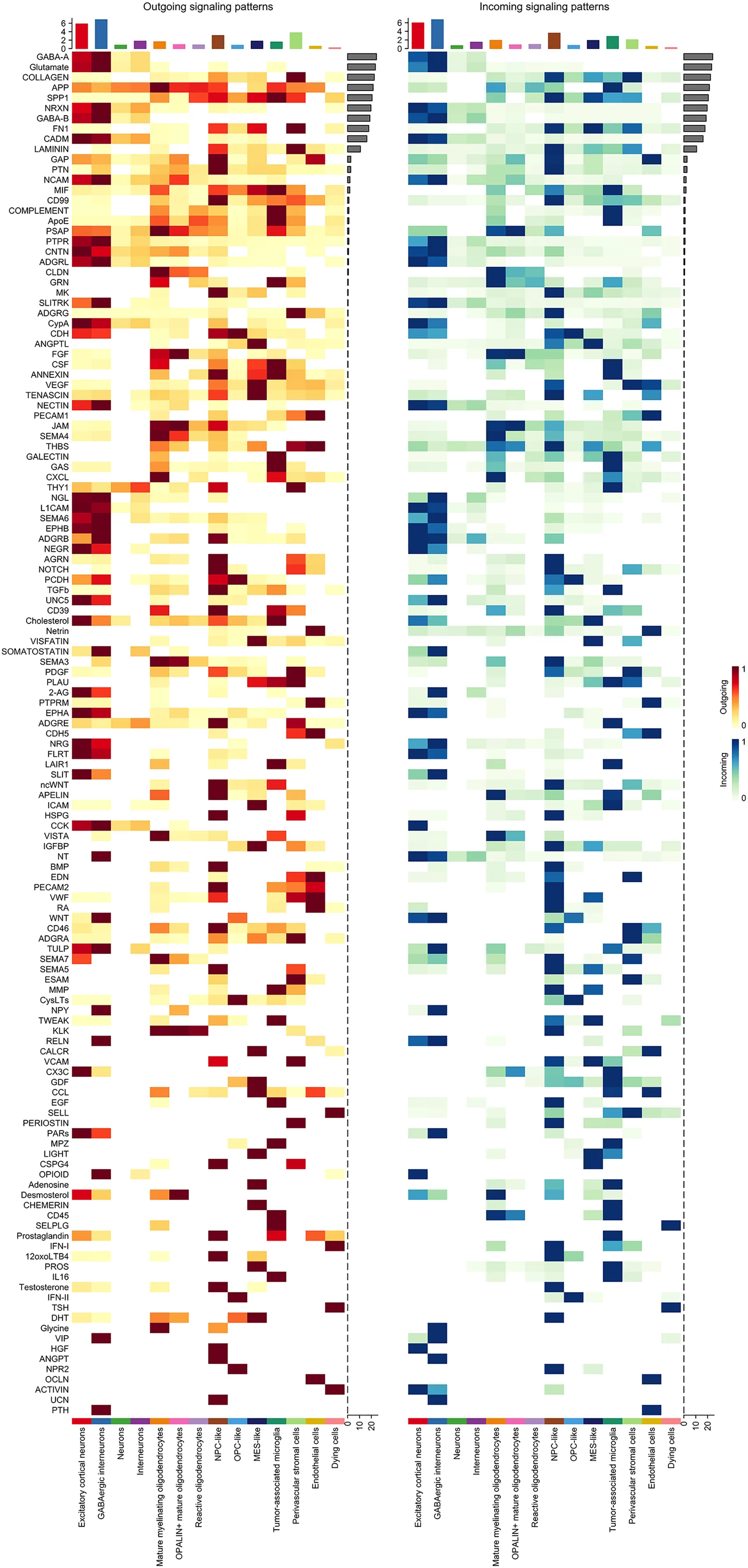
Spatial cell-cell communication among the reconstructed cells (Steps 20–23) CellChat signaling-role heatmaps computed under a 50 μm interaction distance: signaling pathways (rows) across the annotated cell types (columns). Left, outgoing signaling strength (cells acting as senders); right, incoming signaling strength (cells acting as receivers). The colored bars along the top summarize the total signaling strength per cell type, and the gray bars at the right summarize the total strength per pathway.

## Expected outcomes

Running the deconvolution on the example dataset reconstructs approximately 45,530 in-silico single cells from 10,878 Visium spots under an 11-topic model, exported in the standard 10x HDF5 layout. Each cell carries a spatial coordinate within its parent spot and a topic-of-origin label, so the object behaves like a single-cell spatial experiment. The mean occupancy is 4.3 cells per spot, and 363 of the 10,878 spots (3.3%) are called empty and assigned no cells.

Loaded into Seurat with BPCells, the reconstructed cells cluster into transcriptionally coherent populations that map to interpretable regions of the tissue (Figure 6). On this glioblastoma section the annotation resolves fourteen populations spanning the neural, glial, malignant, immune and vascular compartments of the tumor and its margin. In descending size: excitatory cortical neurons (6,580 cells), dying cells (5,475), NPC-like (5,153), GABAergic interneurons (4,294), mature myelinating oligodendrocytes (4,128), OPALIN-positive mature oligodendrocytes (3,388), MES-like (3,308), tumor-associated microglia (3,099), a further neuronal population (2,924), reactive oligodendrocytes (2,220), interneurons (1,908), perivascular stromal cells (1,797), endothelial cells (780) and OPC-like (476). The smallest population is therefore about 1% of the reconstruction, which is the kind of minor population the topic-selection rule of Step 11 is designed to retain.

The recovery of three distinct malignant states alongside three oligodendrocyte states is the outcome to check first, because it is what distinguishes a successful run from an over-merged one. Spot-level clustering of the same section does not separate them: at 55 μm each spot mixes malignant cells with the resident glia they infiltrate, so the spot-level profile is intermediate between the two. Recovering them requires the per-cell reconstruction. If a run returns a single undifferentiated malignant cluster, the topic number is the first thing to revisit.

Standard visualizations confirm the spatial organization. A SpatialDimPlot over the tissue image (Figure 6A) shows that the annotated populations occupy distinct territories rather than being uniformly mixed, and the UMAP of the annotated cells (Figure 6B) shows the malignant states forming a connected continuum, consistent with the transitional nature of glioblastoma cell states, while the neuronal, oligodendrocyte and vascular populations form separated islands. The marker DotPlot (Figure 6D) confirms that each annotated population is defined by a coherent and largely exclusive marker set.

A neighborhood co-localization analysis (Figure 6C) summarizes which cell types are spatial neighbors. The matrix is dominated by a strong diagonal, since cells of the same type frequently share a spot, and the off-diagonal signal resolves into four compartments. The four neuronal populations - excitatory cortical neurons, GABAergic interneurons, neurons and interneurons - form a mutually associated block. The three oligodendrocyte states form a second, with mature myelinating and reactive oligodendrocytes the most strongly linked pair. The malignant states form a third, in which NPC-like and OPC-like are neighbors while MES-like is almost exclusively self-associated. The fourth couples tumor-associated microglia to perivascular stromal cells and those in turn to endothelial cells, reconstructing the perivascular niche; NPC-like cells also associate with both microglia and perivascular stroma, and dying cells with microglia. The neuronal and malignant blocks barely touch, which separates infiltrated cortex from tumor.

The CellChat step yields per-pathway communication networks and, in particular, paired incoming and outgoing signaling-role heatmaps (pathways by cell types) that identify which reconstructed populations act as senders and which as receivers within the recorded interaction radius. On this section 162 of the 182 ordered cell-type pairs have a measurable distance inside the 50 μm radius and 3,071 over-expressed ligand-receptor pairs enter the inference. Ranking pathways by total contribution returns three coherent groups. The strongest are neuronal and synaptic: GABA-A, glutamate, GABA-B, and the synaptic adhesion families NRXN, CADM, NCAM, CNTN and PTPR, which together describe the resident neural tissue at the infiltrated margin. The second group is extracellular matrix - COLLAGEN, FN1 and LAMININ - consistent with the mesenchymal and perivascular compartment. The third is the tumor-microglia axis: SPP1, PTN, MIF, APP, ApoE, PSAP, CD99 and COMPLEMENT.

## Quantification and statistical analysis

Topic-number selection in Steps 10 and 11 uses held-out perplexity on a fixed 70/30 train/test split of spots, evaluated across K, alongside a count of rare topics (mean per-spot theta below perc_rare_thresh). The recommended K is chosen by an explicit rule rather than by inspection: writing P(K) for the held-out perplexity at K over the swept range, the low-perplexity set is the K values whose perplexity falls at or below the k_band quantile of the sweep, and the recommendation is the largest K in that set. With the default k_band = 0.05 this is the 95th-percentile rule, and the cut sits close to the minimum, so the recommendation reads as “among the K values statistically tied with the best fit, take the most resolved one”. Rare topics are retained deliberately rather than avoided, because in a reference-free, cell-generating setting they correspond to minor cell populations recovered as individual cells. Two alternative rules are provided for comparison: a relative-tolerance rule keeping every K within a fraction toll of the best perplexity, and the plain minimum. A recommendation equal to max_k is flagged, since it indicates the sweep did not extend far enough for perplexity to rise again.

Topic alignment across seeds and slices uses Hungarian matching on cosine similarity of topic-gene vectors, and topic stability is quantified as the mean pairwise cosine similarity of aligned topics. The sampling engine conserves per-spot UMIs and draws cells via a batched sequential-binomial multinomial sampler with a fixed seed. Downstream, Seurat marker detection uses Wilcoxon rank-sum tests (only.pos, with min.pct and log-fold-change thresholds as configured), and CellChat computes communication probabilities by a truncated mean over each cell group with spatial distance as a constraint.

## Limitations

CoexpressDeconvolve reconstructs statistically plausible single cells from a mixed spot signal. The in-silico cells are model draws, not physically resolved single cells, and the fine spatial arrangement within a spot is imposed geometrically rather than measured.

Two consequences follow. Neighborhood and communication results are interpreted at the level of co-occupancy of the same capture spot, which is measured, and not at the level of position within a spot; Step 21 enforces this by requiring an interaction range that spans the whole capture-spot footprint. And every later quantity descends from the housekeeping-based cell-density calibration of Step 7, so a section whose housekeeping signal is not comparable to the species standard, heavily degraded material for example, propagates that error to the cell count and to everything derived from it.

## Troubleshooting

### Problem 1

The gene manifold is unannotated, or density calibration fails, for an organism other than human or mouse.

### Potential solution

Generate a species profile with the CoexpressDeconvolve Config Maker (“Estimate single cell parameters.ipynb”): point it at a folder of 10x scRNA-seq datasets for the target organism and set species; it computes hk_profiles and engine_parameters (mu, phi) and writes the species block into codeconv_config.json. Add organism-specific qc_markers and noise_regex to that block so the manifold is annotated. Use codeconv.write_default_config() to obtain a correctly structured file to edit.

### Problem 2

After Step 7, almost all spots collapse to zero or one cell, or too many spots are dropped as background.

### Potential solution

Lower min_umi so that genuine tissue spots are retained, and adjust anchor_mean_factor so the calibration standard line sits within the bulk of the per-spot housekeeping signal. For weak sections set low_slice_quality=True to enforce a one-cell floor and topic rescue.

### Problem 3

Consensus topics for multiple sections look unstable or duplicated across seeds in Step 12.

### Potential solution

Increase the number of consensus seeds (n_seeds) in Step 12 for a more stable consensus and re-inspect topic stability. Only if two topics are genuinely redundant should K be lowered slightly. Do not lower K merely to remove rare or small topics: they are expected and desirable here, because they correspond to minor cell populations recovered as individual cells.

### Problem 4

Seurat::Load10X_Spatial cannot find or read the exported object (Step 16).

### Potential solution

Point Load10X_Spatial at slice_<name>/deconvolved/, which contains filtered_feature_bc_matrix.h5 and spatial/. Confirm that Step 16 completed and that the output_folder path is correct. For a platform with no histology, confirm that the placeholder image was written; the warning printed at export names the canvas size.

### Problem 5

The K-sweep recommendation lands on max_k, or perplexity is still falling at the end of the sweep (Steps 10 and 11).

### Potential solution

This is the one case where the automated rule cannot be trusted, and it is flagged with a warning. The rule returns the largest K in the low-perplexity band, so if perplexity has not yet turned upward, the largest K in the band is simply the largest K that was tried, and the sweep has not bounded K from above. Re-run Step 10 with max_k raised by roughly half, for example from 15 to 30, keeping min_k unchanged so the curves remain comparable, and inspect Figure 5 again. A well-bounded sweep is U-shaped: perplexity falls steeply, flattens, and rises appreciably before max_k. If the curve is still flat at K = 30, the limitation is usually the corpus rather than the range, and the diagnosis is upstream. Check on Figure 3 that the selected genes sit clearly above the mean-variance trend, and consider raising n_odg. A flat perplexity curve across a wide K range means the model gains nothing from additional topics, which happens when the overdispersed-gene set carries little structure. Do not simply accept a large K in this situation: confirm in Step 13 that the additional topics have distinct marker sets rather than near-duplicate ones.

## Resource availability

### Lead contact

Requests for further information and resources should be directed to and will be fulfilled by the lead contact, Olga Perik-Zavodskaia (perik.zavodskaia@gmail.com).

### Technical contact

Technical questions on executing this protocol should be directed to the technical contact, Olga Perik-Zavodskaia (perik.zavodskaia@gmail.com).

### Materials availability

This protocol did not generate new physical materials. It uses a publicly available 10× Genomics dataset.

### Data and code availability

The example dataset is publicly available from 10× Genomics (see key resources table). The CoexpressDeconvolve source code is publicly available on GitHub (https://github.com/Perik-Zavodskii/CoexpressDeconvolve), distributed on PyPI as codeconv (https://pypi.org/project/codeconv/), and archived at Zenodo (https://doi.org/10.5281/zenodo.21860849). Any additional information required to reanalyze the data reported in this protocol is available from the lead contact upon request.

## Acknowledgments

This research was supported by the 10.13039/501100006769Russian Science Foundation, award no. 25-15-00095 (https://rscf.ru/project/25-15-00095/). The authors thank the 10× Genomics team for making spatial and multi-modal datasets publicly available.

## Author contributions

Conceptualization, S.A., R.P.-Z., and O.P.-Z.; methodology, S.A., R.P.-Z., and O.P.-Z.; software, S.A., R.P.-Z., and O.P.-Z.; formal analysis, S.A., R.P.-Z., and O.P.-Z.; data curation, S.A., R.P.-Z., and O.P.-Z.; visualization, S.A., R.P.-Z., and O.P.-Z.; writing – original draft, S.A., R.P.-Z., O.P.-Z., and S.S.; writing – review and editing, S.A., R.P.-Z., O.P.-Z., and S.S.; supervision, S.S.

## Declaration of interests

The authors declare no competing interests.
